# The Correlation of Aortic Neck Angle and Length in Abdominal Aortic Aneurysm with Severe Neck Angulation for Prediction of Intraoperative Neck Complications and Postoperative Outcomes after Endovascular Aneurysm Repair

**DOI:** 10.3390/jcm12185797

**Published:** 2023-09-06

**Authors:** Khamin Chinsakchai, Thana Sirivech, Frans L. Moll, Sasima Tongsai, Kiattisak Hongku

**Affiliations:** 1 Division of Vascular Surgery, Department of Surgery, Faculty of Medicine Siriraj Hospital, Mahidol University, Bangkok 10700, Thailand; dent1234thanatoy@gmail.com (T.S.); kiattisak.hongku@gmail.com (K.H.); 2 Vascular Surgery Department, University Medical Center Utrecht, 3584 Utrecht, The Netherlands; fransmoll50@gmail.com; 3 Research Department, Faculty of Medicine Siriraj Hospital, Mahidol University, Bangkok 10700, Thailand; sasima.ton@mahidol.edu

**Keywords:** abdominal aortic aneurysm, severe neck angulation, neck length, index, outcomes

## Abstract

Objectives: Endovascular aneurysm repair (EVAR) in a hostile neck has been associated with adverse outcomes. We aimed to determine the association of infrarenal aortic neck angle and length and establish an optimal cutoff value to predict intraoperative neck complications and postoperative outcomes. Methods: This was a retrospective review of patients with an intact infrarenal abdominal aortic aneurysm (AAA) with severe neck angulation (>60 degrees) who underwent EVAR from October 2010 to October 2018. Demographic data, aneurysm morphology, and operative details were collected. The ratio of neck angle and length was calculated as the optimal cutoff value of the aortic neck angle-length index. The patients were categorized into two distinct groups using latent profile analysis, a statistical technique employed to identify concealed subgroups within a larger population by examining a predetermined set of variables. Intraoperative neck complications, adjunct neck procedures, and early and late outcomes were compared. Results: 115 patients were included. Group 1 (G1) had 95 patients with an aortic neck angle-length index ≤ 4.8, and Group 2 (G2) had 20 patients with an aortic neck angle-length index > 4.8. Demographic data and aneurysm morphology were not significantly different between groups except for neck length (*p* < 0.001). G2 had more intraoperative neck complications than G1 (21.1% vs. 55%, *p* = 0.005). Adjunctive neck procedures were more common in G2 (18.9% vs. 60%, *p* < 0.001). The thirty-day mortality rate was not statistically different. G1 patients had a 5-year proximal neck re-intervention-free rate comparable to G2 patients (93.7% G1 vs. 87.5% G2, *p* = 0.785). The 5-year overall survival rate was not statistically different (59.9% G1 vs. 69.2% G2, *p* = 0.891). Conclusions: Patients with an aortic neck angle-length index > 4.8 are at greater risk of intraoperative neck complications and adjunctive neck procedures than patients with an aortic neck angle-length index ≤ 4.8. The 5-year proximal neck re-intervention-free rate and the 5-year survival rate were not statistically different. Based on our findings, this study suggests that the aortic neck angle-length index is a reliable predictor of intraoperative neck complications during EVAR in AAA with severe neck angulation.

## 1. Introduction

Endovascular aneurysm repair (EVAR) is now performed in up to 44% of abdominal aortic aneurysm (AAA) surgeries outside the instructions for use, mostly due to hostile aortic neck anatomies [1]. Two of the most important predictors of EVAR failure in AAA are infrarenal aortic neck angulation [2,3] and short aortic neck [4,5], conditions that are associated with a significantly higher rate of early and late-type 1A endoleak.

As per the expert panel’s assessment of EVAR’s hostile neck definition, a severe neck angulation is deemed to be present when the infrarenal neck angulation exceeds 60 degrees, while a neck length of less than 10 mm is classified as a short neck [1].

While some studies have reported good early and mid-term outcomes in AAA with severely infrarenal neck angulation [6,7], this condition has a tendency to develop type 1A endoleaks during long-term follow-up [8]. The correlation between the severely infrarenal neck angulation and neck length for AAA treated with EVAR has not been studied.

Therefore, we investigated whether the index of infrarenal aortic neck angulation and length has any effect on intraoperative complications and outcomes after EVAR. We aimed to determine the association of infrarenal aortic neck angle in severely angulated aortic neck and length to find the optimal cutoff value for prediction of intraoperative neck complications after EVAR and to compare early and late outcomes between groups classified by the aortic neck angle-length index.

## 2. Materials and Methods

### 2.1. Patient Selection

All intact infrarenal AAA patients with severe neck angulation (infrarenal neck angle greater than 60°) who underwent standard EVAR at our institute between October 2010 and October 2018 were enrolled in this study. Patients who lacked preoperative computed tomographic angiography (CTA) data were excluded. The study protocol was approved by the Siriraj Institutional Review Board (SIRB) (COA no. Si 499/2018). Data were collected from a prospectively maintained AAA database through a retrospective review of medical records. Demographic data and clinical-specific profiles were recorded including age, sex, and medical co-morbidities. In addition, the American Society of Anesthesiologists (ASA) physical status classification estimated by an anesthesiologist was also documented.

### 2.2. Preoperative CT Scan Measurement

Preoperative assessment of aneurysm morphology was performed using a multidetector CTA with thin-slice (1 mm) imaging. Accurate measurements were determined using post-processing 3D imaging software, such as Osirix MD 13.0.0 (Pixemo, Geneva, Switzerland). The aortic neck angle and length measurements were determined by two observers (KC and TS). The two investigators performed the angle and length measurements independently and in a random order. Infrarenal aortic neck angulation was defined as the maximal angle from all views between the proximal aortic neck and the longitudinal axis of the aneurysm [9]. Infrarenal aortic neck length was defined as the first diameter showing growth of 10% over the diameter at the most caudal renal artery [10]. A conical neck was defined as a gradual neck dilatation of ≥2 mm within the first 10 mm after the most caudal renal artery [11]. Other AAA morphologies were recorded including maximal diameter, suprarenal neck angulation, ≥50% circumferential proximal neck thrombus (≥2 mm thick), ≥50% calcified proximal neck, and AAA length (the length from the lowest renal artery to aortic bifurcation). For determination of intraobserver reliability, the co-author (TS) measured both the infrarenal aortic neck angle and neck length twice with an interval of 1 to 2 weeks. The aortic neck angle and neck length were measured by two observers (KC and TS) to evaluate the interobserver reliability. Details of these measurements were reviewed separately without the knowledge of the clinical outcomes of the patients to avoid measurement bias.

### 2.3. Definitions and Outcomes Measurement

**Figure 1:**
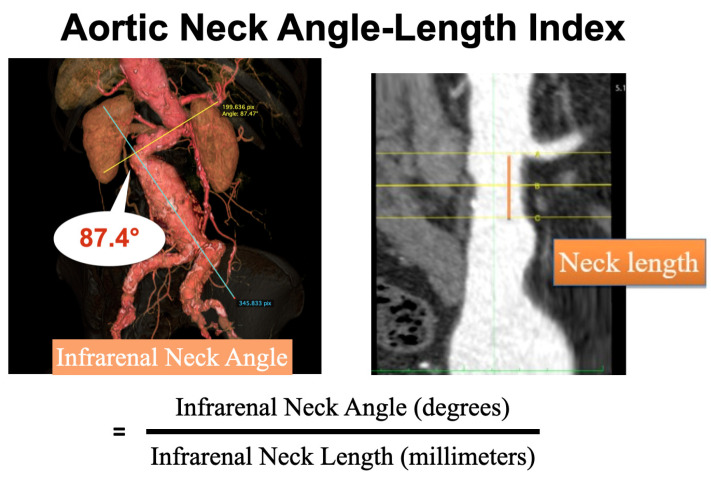
How to calculate an optimal cutoff value of the aortic neck angle-length index.

The aortic neck angle-length index was calculated using the average of the infrarenal neck angle (degree) divided by the average of the infrarenal aortic neck length (millimeter) (Figure 1). Intraoperative neck complications were detected by completion angiography after the endografts were deployed completely. We focused on four types of intraoperative neck complications. A type 1A endoleak was defined as the extravasation of contrast between the prosthesis and aneurysm wall from the proximal neck [12]. Endograft migration was determined by the displacement of the stent graft caudally, measuring the distance from the lowest renal artery and the most cephalad portion of the stent graft > 10 mm [12]. Renal artery occlusion was defined as partial or complete occlusion of one or bilateral renal arteries. Aortic dissection was determined as retrograde aortic dissection.

Intraoperative adjunctive neck procedure was defined as any other procedure designed to augment the effects of the principal procedure, especially for management of intra-operative neck complications or otherwise unsatisfactory outcomes such as proximal extension cuff and Palmaz stent placement (Cordis Corp., Miami Lakes, FL, USA). Operative details were composed of the brand of aortic stent graft product, procedure time (minutes), fluoroscopic time (minutes), volume of contrast usage (milliliters), and intraoperative blood loss (milliliters).

In-hospital death or any death or complications occurring during the first 30-day postoperative period were analyzed. Complications were defined according to a previous report by Chaikof et al. [12].

All patients undergoing EVAR were followed according to a predetermined protocol that included a CTA at 1, 6, and 12 months, and then every 12 months thereafter. In patients with stage IV or V chronic kidney disease (eGFR < 30 mL/min per 1.73 m^2^), the aortic stent graft was evaluated via standard duplex ultrasound performed by a radiologist. All radiologic exams conducted after endovascular repair were reviewed for migration, endoleaks, graft limb occlusions, aneurysm sac size, and re-interventions.

### 2.4. Statistical Analysis

The required sample size for point biserial correlation was conducted in G*Power 3.1.9.2 using a significance level of 0.05, a power of 0.90, a medium effect size (ρ = 0.3), and a two-tailed test. Given the absence of a definitive rule for determining sample size in latent profile analysis, Wurpts et al. [13] have recommended a minimum of 100 subjects as a reasonable sample size. Consequently, all subjects meeting the inclusion criteria were recruited. Building upon this assumption, the desired sample size for our study was determined to be 109.

Data were recorded and analyzed using PASW Statistics 18.0 (SPSS Inc., Chicago, IL, USA). Descriptive statistics were used to describe demographic and clinical characteristics. For normal distribution data, quantitative variables were described using mean and standard deviation (SD), while non-normal distribution data were described using median and range. Qualitative data were expressed by number and percentage. An intraclass correlation coefficient (ICC) was used to evaluate intraobserver reliability using a two-way mixed effects model and interobserver reliability using a two-way random effects model. A latent profile analysis (LPA) was used to classify groups of patients based on aortic neck angle-length index using the R package mclust [14]. LPA is a method that aims to identify latent subpopulations within a larger population by analyzing a specific set of variables. LPA assumes that individuals can be classified into different categories with varying probabilities, each characterized by unique combinations of personal and/or environmental attributes [15]. Four indices were used to select the correct number of latent classes, i.e., log-likelihood, Bayesian information criterion (BIC), integrated complete-data likelihood, and bootstrap likelihood ratio test (BLRT). Lower BIC and integrated complete-data likelihood values coupled with higher log-likelihood values show and indicate a better-fitting model. The BLRT was used to compare successive models, where a significant change in −2 log-likelihood indicated that the model with the greater number of classes provided a better fit to the data. Point biserial correlation coefficient (*r*_pb_) was used to examine the relationship between the aortic neck angle-length index score and two classes of aortic neck angle-length groups classified by LPA. In comparison between two groups, the Pearson chi-square test, Yates’ continuity correction, or Fisher’s exact test was performed for qualitative variables, and the independent sample t-test or Mann-Whitney U test was used for quantitative variables with means or medians, as appropriate. The Kaplan-Meier method was performed to estimate overall survival time and re-intervention free time. The log-rank test or Breslow test was used to compare overall survival and re-intervention free time between aortic neck angle-length index groups. All tests were considered statistically significant with a *p*-value less than 0.05.

## 3. Results

From October 2010 to October 2018, 759 patients were diagnosed with intact AAA at our institution. Open surgical repair, fenestrated or chimney EVAR procedure, and AAA without severely angulated infrarenal neck were excluded. One hundred and fifty-five of these patients presented with a severely angulated neck of AAA treated with EVAR. Forty patients were not included in this study due to lack of preoperative CTA (*n* = 30), using other brands of aortic stent graft (Nelix, AFX, Ovation, and Gore Excluder, *n*= 7), using funnel technique (*n* = 2), and previous open surgical repair (*n* = 1). In total, 115 patients were enrolled in our study.

### 3.1. Intraobserver and Interobserver Reliability

Intraobserver reliability of the author on aortic neck length had an ICC of 0.988 (95% CI; 0.982–0.992) and infrarenal angle showed excellent reliability with an ICC of 0.986 (95% CI; 0.981–0.991). Interobserver reliability of two observers on neck length showed excellent and moderate reliability with an ICC of 0.969 (95% CI; 0.954–0.979) and aortic neck angle with an ICC of 0.739 (95% CI; 0.632–0.816).

### 3.2. Aortic Neck Angle-Length Index

The aortic neck angle-length index was calculated from the average of the infrarenal neck angle (degree) divided by the average neck length (millimeter) for each patient (Figure 1). The median of the aortic neck angle-length index was 3.2 (range 1.1–14.3). LPA classified the aortic neck angle-length index into two groups, i.e., aortic neck angle-length index ≤ 4.8 [Group 1, (G1)] and aortic neck angle-length index > 4.8 [Group 2, (G2)], which represented 95 (82.6%) and 20 (17.4%) patients, respectively. The relationship between the aortic neck angle-length index score and two classes of aortic neck angle-length groups was high with *r*_pb_ of 0.780 (95% CI: 0.704–0.857). There were no statistically significant differences in demographic variables (Table 1). Most of the patients were male (70.5% G1 and 80% G2), and the mean patient age was 77.1 ± 6.4 (G1) and 75.7 ± 7.2 (G2) years, respectively. Hypertension was the most common comorbidity, presented in almost three-quarters (80% G1 and 75% G2), followed by dyslipidemia (45.3% G1 and 40% G2).

### 3.3. AAA Morphology

All patients had a fusiform-type aneurysm. There were no statistically significant differences in the median of the suprarenal neck angle (43.6° G1 vs. 40.5° G2, *p* = 0.912) or the mean of the infrarenal neck angle (82.9° G1 vs. 89.7° G2, *p* = 0.108). The aortic neck length was significantly shorter in Group 2 (34.1 mm vs. 14.0 mm, *p* < 0.001). The other aneurysm morphologies were not statistically significantly different (Table 2).

### 3.4. Intraoperative Outcomes

For intraoperative outcomes (Table 3), Group 2 had significantly higher intraoperative neck complication rates than Group 1 (21.1% G1 vs. 55% G2, *p* = 0.005), especially type IA endoleaks (10.5% G1 vs. 40% G2, *p* < 0.003). There was no significant difference in endograft migration and renal artery coverage between the two ANAL index groups.

In Group 2, the adjunctive neck procedures were performed significantly more frequently (18.9% G1 vs. 60% G2, *p* < 0.001). The proximal extension cuff placements were more common in Group 2 (7.4% G1 vs. 25% G2, *p* = 0.034), and Palmaz stent placements were more common in Group 2, but the difference was not statistically significant (9.5% G1 vs. 20% G2, *p* = 0.237).

### 3.5. Operative Details

The Endurant, Zenith, and Treovance devices were used to perform EVAR, and there were no statistically significant differences between the two groups. The mean of procedure time (minutes), fluoroscopic time, volume of contrast usage, and intraoperative blood loss were all higher in the aortic neck angle-length index > 4.8 (G2), but these did not reach statistical significance. Operative details are summarized in Table 3.

### 3.6. Early Postoperative Outcomes

There was no statistically significant difference in 30-day complications (30.5% G1 vs. 40% G2, *p* = 0.575) or in deployment-related complications (6.3% G1 vs. 10% G2, *p* = 0.626). In eight patients with deployment-related complications, two aortic dissections occurred within 30 days. One patient required open repair. Four patients had access site hematoma, and one required surgical evacuation. One patient had distal embolization, treated with transfemoral thromboembolectomy and another patient had a dissection of the right external iliac artery with conservative treatment.

There was one procedure-related death in Group 1 from a retrograde aortic dissection on postoperative day 8, and one patient in Group 2 died from severe colonic ischemia on postoperative day 2. There was no statistically significant difference in the 30-day mortality rate between the two groups (1.1% G1 vs. 5% G2, *p* = 0.319). Two patients in Group 1 died after 30 days, one from pulmonary infection with severe sepsis and another from multiple organ failure. One patient in Group 2 died from pulmonary infection with severe sepsis on postoperative day 85 (3.1% G1 vs. 10% G2, *p* = 0.439). The median length of stay was not statistically significantly different between the two groups (7 days, G1 vs. 8 days, G2, *p* = 0.401). Post-operative outcomes are shown in Table 4.

### 3.7. Late Outcomes

**Figure 2:**
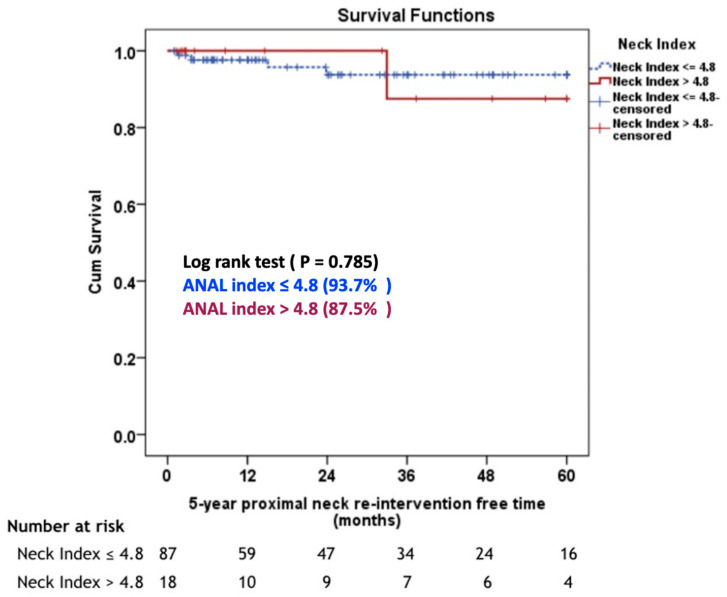
Five-year proximal neck re-intervention-free rate between 2 groups of aortic neck angle-length indexes. Abbreviation: ANAL, aortic neck angle-length index.

**Figure 3:**
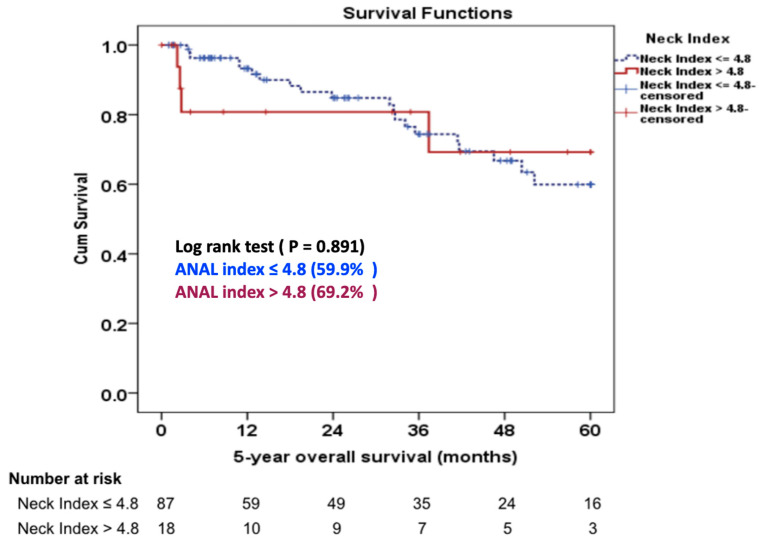
Five-year overall survival rate between aortic neck angle-length indexes groups. Abbreviation: ANAL, aortic neck angle-length index.

The median follow-up duration was 36.6 months (range: 1–110 months). During the 6-month follow-up, one case experienced graft limb occlusion which was subsequently managed through a femorofemoral polytetrafluoroethylene (PTFE) bypass graft procedure. In the 5-year proximal neck re-intervention-free rate, there was no statistically significant difference between the aortic neck angle-length index ≤ 4.8 group and the aortic neck angle-length index > 4.8 group (93.7% G1 vs. 87.5% G2, *p* = 0.785), (Figure 2). Four (4.2%) patients in Group 1 required proximal neck re-intervention due to a type 1A endoleak (*n* = 3) and a type 3B endoleak (*n* = 1). One patient had a type 3B endoleak at 15 months postoperatively at the main body of the stent graft due to the pressure effect of the Palmaz stent and was treated successfully with a proximal extension cuff. Three patients developed a type 1A endoleak during follow-up. One patient required an aortic extension cuff at four months and two patients needed a Palmaz stent at two and 24 months, respectively. In Group 2, one (5%) patient underwent a proximal extension cuff placement due to a ruptured AAA from a type 1A endoleak at 33 months postoperatively. There was no statistically significant difference in the 5-year overall survival rate between the two groups (59.9% G1 vs. 69.2% G2, *p* = 0.337), (Figure 3).

## 4. Discussion

The suitability of EVAR is usually based on the manufacturer’s instructions for use. Strict adherence to the standard requirements generally leads to good outcomes. EVAR within a hostile neck anatomy, particularly in short and severe neck angulation, is associated with poor early and late outcomes [3,5]. One study of AAA with severely angulated necks revealed significant changes in velocity, flow streamline, pressure, and wall shear stress inside the aneurysm sac [16]. These findings provide valuable insights that can help predict the occurrence of ruptured AAA. We measured a correlation between aortic infrarenal neck angulation and aortic neck length, showing a higher rate of intraoperative neck complications and immediate adjunctive neck procedures in patients with an aortic neck angle-length index > 4.8. Nevertheless, there was no statistically significant difference in the rate of re-intervention or survival between the two groups at late follow-up.

The success of EVAR is largely dependent on the ability of the device to achieve proper fixation and seal [17]. However, inadequate fixation and seal, particularly at the proximal neck, can pose a significant risk of type 1A endoleaks and subsequent rupture of the AAA following EVAR. Researchers have conducted studies to explore predictive factors for AAA rupture. One study found that specific imaging markers obtained from CTA and characteristics of intraluminal thrombus morphology can be used to assess the risk of AAA rupture [18]. Another study focused on geometric parameters, such as the proximal neck angle, which is associated with aortic wall stress, as potential predictors of AAA rupture risk [19]. Additionally, research has shown that a neck length of <15 mm is associated with higher rates of early and late proximal type 1A endoleaks [5], while severe infrarenal neck angulation increases the likelihood of early type 1A endoleaks and graft migration [3]. Similarly, EVAR performed in the presence of a hostile neck morphology, characterized by a neck length of <10 mm and infrarenal neck angulation >60 degrees, has been found to be associated with a higher rate of intraoperative type 1A endoleaks and immediate adjunct neck procedures compared to cases with favorable aortic neck anatomy [11].

Our study provided additional insight into the effect of the aortic neck length and angulation on EVAR outcomes. Consistent with a recent meta-analysis, we found that patients with an aortic neck angle-length index > 4.8 (G2) have higher rates of immediate adjunct neck procedures in short aortic neck length < 15 mm, and/or neck angulation > 60 degrees [20]. No study has reported this association or proposed an optimal cutoff value of the aortic neck angle-length index for the prediction of intraoperative neck complications and immediate adjunct neck procedures after EVAR in AAA with severe neck angulation.

We found that patients with a short neck and an aortic neck angle-length index > 4.8 had significantly higher intraoperative neck complication rates (21% G1 vs. 55% G2, *p* = 0.005) and immediate adjunct neck procedures (18.9% G1 vs. 60% G2, *p* < 0.001). All patients in both groups with intraoperative type 1A endoleaks were successfully treated with a proximal extension cuff and/or Palmaz stent placement to achieve a proximal seal. This finding is consistent with the meta-analysis by Antoniou et al. [20] where adjunctive neck procedures were needed in 22% of AAA with hostile neck anatomy. In addition, short aortic neck length may be a crucial factor in determining neck complications and the requirement for adjunct neck procedures during EVAR in AAA with a severely angulated neck [5,21]. The findings of intraoperative neck complications in our study also support the repair of type 1A endoleaks with a large balloon-expandable Palmaz stent that Cox et al. [22] reported to be beneficial for use in hostile aortic neck, including proximal aortic neck angle > 60 degrees. Despite the danger of type 1A endoleaks, some studies [23,24] have suggested conservative management only in patients with anatomy considered suitable for EVAR, and if an adequately oversized stent graft had been optimally deployed. All type 1A endoleaks in our study developed in patients with severely angulated necks; therefore, we usually performed adjunct neck procedures for immediate type 1A endoleak repair.

Procedure time, fluoroscopic time, volume of contrast usage, and intraoperative blood loss were all higher in the aortic neck angle-length index > 4.8 group (G2), but without reaching statistical significance, perhaps due to an insufficient number of patients. Patients with aortic neck angle-length index > 4.8 (G2) had higher rates of intraoperative neck complications and needed more adjunctive neck procedures. Therefore, the procedure time, fluoroscopic time, contrast usage, and blood loss were higher than in the aortic neck angle-length index ≤ 4.8 (G1). A recently published study has also reported increases in these operative details in the presence of hostile neck anatomy [21].

No statistically significant difference in perioperative mortality was found between the two groups (1.1% G1 vs. 5% G2, *p* = 0.319), which agrees with earlier reports on hostile neck anatomy leading to 30-day mortality between 1.8% and 3% [11,25]. Within our study, there was an occurrence of graft limb occlusion in a single patient, which necessitated the implementation of a femorofemoral PTFE bypass graft procedure. In a separate investigation conducted by Catanese et al. [26], the incidence of limb graft occlusion following EVAR was reported to be 2.5% at a median postoperative day of 27. We observed that freedom from five-year re-intervention was in 93.7% of patients in the aortic neck angle-length index ≤ 4.8 (G1), and in 87.5% of the aortic neck angle-length index > 4.8 (G2), (*p* = 0.785). This is consistent with a study by Oliveira et al. [7] who reported a 95% freedom from four-year re-intervention in severely angulated neck and with Aburahma et al. [11] who reported an 85% freedom from four-year re-intervention in hostile aortic neck anatomy. The estimated overall survival rate at five years was 59.9% in patients with the aortic neck angle-length index ≤ 4.8 (G1), and 69.2% in the aortic neck angle-length index > 4.8 (G2), respectively (*p* = 0.337). Surprisingly, few studies have addressed long-term survival in AAA with severely angulated neck. Oliveira et al. [8] described 7-year survival rates of 44.3% for AAA with severely angulated neck.

Our study has certain limitations that should be acknowledged. Firstly, the small sample size, retrospective design, and observational nature of the study introduce inherent limitations, such as potential selection bias and reliance on existing data. This may affect the generalizability of our findings. Moreover, the inability to control for confounding variables may impact the validity of the observed associations. Additionally, it is important to note that not all patients included in the study had preoperative CT scans available, leading to potential information gaps. Furthermore, the utilization of different endograft devices for EVAR introduces a potential source of variability in our results. Lastly, it is worth mentioning that the study assessed outcomes only up to a 5-year follow-up period. As a result, the long-term effects and complications associated with EVAR in patients with severe neck angulation may not have been fully captured. A more comprehensive understanding of post-operative outcomes could be obtained by extending the follow-up duration.

## 5. Conclusions

Patients with an aortic neck angle-length index > 4.8 had a higher risk of intraoperative neck complications and adjunctive neck procedures compared with patients with an aortic neck angle-length index ≤ 4.8. The 5-year proximal neck re-intervention-free rate and the 5-year overall survival were not statistically different between groups. The aortic neck angle-length index is a reliable predictor of intraoperative neck complications and to prepare backup devices for immediate adjunct neck procedures during EVAR in AAA with a severely angulated neck.

## Figures and Tables

**Table 1:** Demographic data and clinical characteristics.

Baseline Characteristics	Group 1 ANAL Index ≤ 4.8 (*n* = 95)	Group 2 ANAL Index > 4.8 (*n* = 20)	*p* Value
Age, mean ± SD years	77.1 ± 6.4	75.7 ± 7.2	0.360
Male gender, no. (%)	67 (70.5%)	16 (80%)	0.559
Cardiac disease, no. (%)	31 (32.6%)	4 (20%)	0.396
COPD, no. (%)	14 (14.7%)	1 (5%)	0.463
Hypertension, no. (%)	76 (80%)	15 (75%)	0.762
Dyslipidemia, no. (%)	43 (45.3%)	8 (40%)	0.885
Chronic kidney disease, no. (%)	23 (24.2%)	2 (10%)	0.235
Diabetes mellitus, no. (%)	14 (14.7%)	1 (5%)	0.463
Cerebrovascular disease, no. (%)	13 (13.7%)	1 (5%)	0.458
ASA class II, no. (%)	22 (23.2%)	6 (30%)	0.674
ASA class III, no. (%)	71 (74.7%)	14 (70%)	0.674

Abbreviation: ANAL, aortic neck angle-length index; no., number; SD, standard deviation; COPD, chronic obstructive pulmonary disease, ASA, American Society of Anesthesiologists.

**Table 2:** Morphology of abdominal aortic aneurysm by aortic neck angle-length indexes.

Aneurysm Morphology	Group 1 ANAL Index ≤ 4.8 (*n* = 95)	Group 2 ANAL Index > 4.8 (*n* = 20)	*p* Value
AAA morphology, fusiform (%)	95 (100%)	20 (100%)	1.000
AAA length (mm), mean ± SD	127.7 ± 24.8	134.2 ± 24.6	0.285
AAA max. diameter (mm), mean ± SD	64.5 ± 14.6	66.6 ± 12.0	0.550
Suprarenal neck angle (°), median (min, max)	43.6 (0, 133.7)	40.5 (13.5, 91.3)	0.912
Infrarenal neck angle (°), mean ± SD	82.9 ± 17.1	89.7 ± 15.9	0.108
Neck length (mm), mean ± SD	34.1 ± 13.8	14.0 ± 3.7	<0.001
Neck calcification, no. (%)	4 (4.2%)	0 (0%)	1.000
Neck thrombus, no. (%)	6 (6.3%)	2 (10%)	0.626
Neck morphology, no. (%)			0.751
Cylindrical	74 (77.9%)	14 (70%)	
Conical	14 (14.7%)	4 (20%)	
Reverse conical	7 (7.4%)	2 (10%)	

Abbreviation: AAA, abdominal aortic aneurysm; ANAL, aortic neck angle-length index; mm, millimeter; no., number; SD, standard deviation.

**Table 3:** Intraoperative details and outcomes between 2 groups of aortic neck angle-length indexes.

Variable	Group 1 ANAL Index ≤ 4.8 (*n* = 95)	Group 2 ANAL Index > 4.8 (*n* = 20)	*p* Value
**Stent graft product, no. (%)**			
Endurant	52 (54.7%)	11 (55%)	0.986
Zenith	39 (41.1%)	8 (40%)	0.986
Treovance	4 (4.2%)	1 (5%)	0.986
**Intraoperative neck complications no. (%)**	20 (21.1%)	11 (55%)	0.005
Type IA endoleak	10 (10.5%)	8 (40%)	0.003
Endograft migration Type IA endoleak and endograft migration	4 (4.2%) 4 (4.2%)	1 (5%) 2 (10%)	1.000 0.279
Renal artery coverage	2 (2.1%)	0 (0%)	1.000
**Adjunctive neck procedures, no. (%)**	18 (18.9%)	12 (60%)	<0.001
Aortic cuff	7 (7.4%)	5 (25%)	0.034
Palmaz stent	9 (9.5%)	4 (20%)	0.237
Aortic cuff and Palmaz stent	2 (2.1%)	3 (15%)	0.036
**Operative details**			
Procedure time, mean ± SD (min.)	178.1 ± 63.5	206.0 ± 75.2	0.087
Fluoroscopic time (min.), median (min, max)	34.6 (15, 110)	40.1 (19, 97)	0.248
Volume of contrast usage (mL), median (min, max)	122 (28, 300)	142.5 (54, 470)	0.162
Blood loss (mL), median (min, max)	250 (50, 1500)	352.5 (75, 2000)	0.082

Abbreviation: ANAL, aortic neck angle-length index; mL, milliliter; no., number; SD, standard deviation.

**Table 4:** Early postoperative outcomes between aortic neck angle-length indexes groups.

Post-Operative Outcomes	Group 1 ANAL Index ≤ 4.8 (*n* = 95)	Group 2 ANAL Index > 4.8 (*n* = 20)	*p* Value
30-day complication, no. (%)	29 (30.5%)	8 (40%)	0.575
Myocardial infarction	0 (0%)	1 (5%)	0.174
Congestive heart failure	3 (3.2%)	1 (5%)	0.540
Respiratory failure	2 (2.1%)	2 (10%)	0.139
Renal failure	5 (5.3%)	1 (5%)	1.000
Deployment-related complications, no. (%)	6 (6.3%)	2 (10%)	0.626
30-day mortality, no. (%)	1 (1.1%)	1 (5%)	0.319
In-hospital mortality *, no. (%)	3 (3.1%)	2 (10%)	0.439
Length of stay, (days) Median (min, max)	7 (2, 124)	8 (2, 85)	0.401

* In-hospital mortality was defined as death occurring during the hospital stay including 30-day mortality.

## Data Availability

Data is unavailable due to privacy or ethical restrictions.
